# Neuropsychological Research in Obsessive-Compulsive Disorder: Current Status and Future Directions

**DOI:** 10.3389/fpsyt.2021.721601

**Published:** 2021-11-01

**Authors:** Himani Kashyap, Amitai Abramovitch

**Affiliations:** ^1^Department of Clinical Psychology, National Institute of Mental Health and Neuro Sciences, Bangalore, India; ^2^Department of Psychology, Texas State University, San Marcos, TX, United States

**Keywords:** cognitive function, neuropsychology, obsessive-compulsive disorder, ecological validity, neurocognitive

## Abstract

Neuropsychological functions in obsessive-compulsive disorder (OCD) have been extensively investigated. Despite some common findings across studies indicating deficient test performance across cognitive domains with small to medium effect sizes, results remain inconsistent and heterogeneous. However, multiple past attempts to identify moderators that may account for such variability have been unrewarding. Typical moderators including symptom severity, age at onset, medication status, and comorbid conditions failed to provide sufficient explanatory power. It has then been posited that these inconsistencies may be attributed to the inherent heterogeneous nature of the disorder (i.e., symptom dimensions), or to the natural fluctuation in symptom severity. However, recent meta-analyses suggest that these factors may not account for the persistent unexplained variability. Other potential factors—some of which are unique to neuropsychological testing—received scarce research attention, including definition of cognitive impairments, specificity and selection of test and outcome measures, and their limited ecological validity. Other moderators, particularly motivational aspects, and metacognitive factors (e.g., self-efficacy) were not previously addressed despite their potential association to OCD, and their documented impact on cognitive function. The aim of the present mini-review is to provide an updated succinct overview of the current status of the neuropsychological literature in OCD and expanding upon oft-neglected potential moderators and their putative impact on neuropsychological findings in OCD. Our goal is to highlight important avenues for further research and provide a road map for investigators in order to advance our understanding of cognitive functions in OCD that has been stagnant in the past decade.

## Introduction

### Neuropsychological Findings in OCD

Decades of research into cognitive function in Obsessive-Compulsive Disorder (OCD), including a number of systematic reviews and meta analyses (1–6), reveal deficient test performance across multiple cognitive domains. Although all meta-analyses consistently report underperformance with small to medium effect sizes in OCD compared to non-clinical controls (see Table 1), a hallmark finding in this literature is significant heterogeneity and inconsistency across studies.

**Table 1:** Unweighted mean effect sizes for neuropsychological test performance across domains in adult OCD.

**Neuropsychological domain/subdomain**	**Unweighted mean ES^a^**	**Magnitude ES^b^**	**Referenced studies**	**Tests included in ES calculations**				
				**Abramovitch et al. (2)**	**Shin et al. (5)**	**Snyder et al. (4)**	**Fradkin et al. (7)**	**Henry (8)**
***Executive Function***								
Set shifting/flexibility	0.42	Small	(2, 4, 5, 7, 8)	CANTAB set shifting, OAT, WCST, TMTB, WAIS similarities	IED, OAT Pers, TMTB, WCST	ID/ED, OAT Pers, DAT, TMTB, WCST, cued task switching	WCST, IDED, TMTB, TS	WCST
Inhibitory function	0.49	Small	(2, 4, 5)	CPT, GNG, SST, Stroop	Stroop	Stroop, SST, GNG		
Working memory	0.33	Small	(2, 4, 5, 7)	CANTAB pattern recognition, CANTAB spatial recognition, CANTAB spatial span, CANTAB SWM, N-Back, WAIS-DS, WMS LNS, WMS spatial span	SWM, WAIS-DS	N-Back, WAIS-DS	OAT, DAT	
Fluency	0.38	Small	(5, 8)		Design fluency, Verbal fluency	Verbal fluency		Verbal fluency
Planning	0.59	Medium	(2, 4, 5)	TOH, TOL	TOH, TOL	TOH, TOL		
***Memory***								
Verbal memory	0.39	Small	(2, 5)	RAVLT, CVLT, AVLT, WMS LM	LM II, VLT			
Non-verbal memory	0.75	Medium	(2, 5)	BVRT, CANTAB pattern recognition, ROCF	RCFT			
***Processing Speed***	0.48	Small	(2, 5)	CPT RT, Choice reaction task, GNG, RT, SST-RT, Stroop congruent RT, TMTA, WAIS SD	TMTA			
***Attention***	0.48	Smal	(2, 5)	CPT, GNG	CPT			
***Visuospatial Function***	0.40	Small	(2, 5)	ROCF copy, Block design	Block design			

*OCD, obsessive-compulsive disorder; ES, effect size; IED, intra/extra dimension; WCST, Wisconsin card sorting test; TMTB, trail making test, part B; OAT, object alternation test; DAT, Delayed alternation test; ID/ED, intradimensional/extradimensional; CANTAB, cambridge automated neuropsychological test battery; SST, stop signal task; OAT Pers., OAT perseverations; DAT; RAVLT, Rey auditory verbal learning test; GNG, go/no-go; AVLT, auditory verbal learning test; CVLT, California verbal learning test; WMS, Wechsler memory scale; LNS, WMS letter number sequencing; BVRT, Benton visual retention scale; ROCF, Rey-Osterrieth complex figure; WAIS, Wechsler adult intelligence scale; DS, WAIS digit span; SD, WAIS symbol digit; SWM, spatial working memory; TOH, tower of Hanoi; TOL, tower of London; CPT, continuous performance test; RT, reaction time; TMTA, trail making task, part A; TS, task switching task; IDED, intra-dimensional/extra-dimensional task; TMT, trail making test; Stroop, Stroop color-word, CBT, Corsi block-tapping test; LM, WAIS logical memory immediate; LM-II, WAIS LM delayed; VLT, verbal learning test*.

a *Unweighted mean effect sizes calculated from the following meta-analyses: (2, 4, 5, 7, 8)*.

b *According to Cohen (9)—positive ES exemplify poorer test performance*.

Indeed, a recent meta-analysis of familial cognitive endophenotypes in OCD also found significant heterogeneity across major executive functions (6). Such inconsistencies suggest that some moderators or latent factors may explain this heterogeneity. However, moderator analyses examining multiple potential variables, including demographic (e.g., sex, age, education) and clinical variables (e.g., age of onset, OCD symptom-severity, medication, comorbidities) found no meaningful moderation effects (2, 4, 5). Moreover, although moderator analyses in meta-analytic reviews usually utilize a meta-regression procedure, some meta-analyses endeavored to examine such potential moderators as the primary outcome. However, these studies, including examinations of correlations between cognitive function and symptom severity (10), and with OCD dimensions (11, 12), found no meaningful effects accounting for such heterogeneity. Moreover, this inconsistency is further obfuscated by research and meta-analysis in pediatric OCD yielding a substantially divergent picture compared with adult OCD (13). Of note, similar extent of heterogeneity has been reported in a meta-analysis examining cognitive functions across studies utilizing the same tasks and outcome measures (as opposed to calculating domain effect sizes from different tests) (5).

Notably, the magnitude of these effects (small to medium) in OCD do not amount to what is typically considered a cognitive *impairment* (2). It is also important to note that the pattern of cognitive dysfunction is not specific to OCD, and a recent umbrella review did not identify any viable disorder-specific biological or cognitive markers for OCD (14). Moreover, similar effect sizes and somewhat similar heterogeneity trends were recently identified across DSM disorders (15–18). This lead to the conclusion that the C Factor (i.e., cognitive dysfunction) is transdiagnostic, and that there is no reliable disorder-specific neuropsychological profile (19). Considering that OCD is associated with functional impairments (20–22), this state of affairs raises the question of whether OCD is linked to meaningful cognitive deficits at all, and if not, whether neuropsychological tests may be poor predictors of everyday functional impairment in OCD. In this review, we outline under-researched factors and several potential latent constructs that ought to be investigated in order to promote our understanding of neuropsychological findings in OCD. These include factors associated with psychometric and interpretive aspects of neuropsychological testing, and state/trait structures associated with OCD or with psychopathology in general.

## Methodological and Psychometric Issues

### Test Selection

One major factor contributing to neuropsychological heterogeneity in OCD is the utilization of different tests under the same general neuropsychological domain. This problem is seen across populations, where different tests assessing a general cognitive domain often yield different results (3). Indeed, researchers have been sounding the alarm about this issue for two decades (1, 23). This problem is evidenced for example in the context of inhibitory function—the most widely researched cognitive domain in OCD. Given the hypothesis that people with OCD struggle to inhibit their urge to perform compulsions, cognitive and behavioral inhibitory dysfunction has been subject to much interest from researchers, and at one point was proposed as an endophenotypic marker for OCD (24). However this was later largely recanted by the authors (25). Notwithstanding, the general domain of inhibitory function is commonly assessed using a number of tests, primarily the Stop Signal Task (SST), the Stroop test, and Go/No-Go/Continuous Performance Tests. However, these tests yield different effect sizes in OCD (2), which may not be surprising because they measure different subdomains of inhibitory function and are associated with different neuroanatomical and neurochemical processes (26–28). Whereas, the Stroop test assesses interference control, the Go/No-Go paradigm assesses response inhibition (inhibition of prepotent motor ‘program’), and the SST assesses response cancellation (29). Since most studies use these tests interchangeably to measure “response inhibition,” the heterogeneity of effect sizes under this construct may be to some extent, a result of problematic conceptualization of such studies, and not a characteristic of OCD. The same problem arises in the context of other neuropsychological domains, including, but not limited to, other executive functions. Unfortunately, despite the decades-old calls to increase precision in test selection and construct definitions (in neuropsychological research in general, as well as specifically in OCD), this problem is still evident in OCD research. This may be a contributing factor to the longstanding issue of unexplained heterogeneity. It is therefore important that neuropsychological studies in psychiatry/psychopathology research involve neuropsychologists, with a careful consideration of underlying constructs, task impurity, psychometrics, and ecological validity (30).

### Selection of Outcome Measures

The ‘task impurity problem’ in neuropsychology is a longstanding issue inherent to cognitive testing, where a several interrelated but distinct cognitive demands are reflected in a single test score (31). This problem, characteristic of most cognitive tests, but more so in tests assessing higher order executive function, poses an interpretive hurdle (32). Several solutions to mitigate this problem have been offered such as utilizing and cross-referencing from more than one test to assess an executive function (33). Carefully attending to the construct validity of specific outcomes within a test should be standard practice in neuropsychological research. For instance, the Wisconsin Card Sorting Test (WCST) is frequently used to assess cognitive flexibility, set-shifting, and concept formation, but performance on the WCST also requires working memory, attention, as well as planning, strategizing, inhibitory control, feedback processing, rule extraction, and self-monitoring (7, 34, 35). Indeed, a recent meta-analysis (7) examined the notion that OCD is associated with deficits in flexibility/set shifting (36)—constructs known for their heterogeneity in OCD—by parceling out different cognitive processes from the same tasks. Differentiating performance on shifting vs. “control” (i.e., non-shifting) outcome measures from the same tests, the authors found no evidence for such deficits in OCD (7). Thus, together with the need to carefully select neuropsychological tests to assess specific domains of cognitive function, an even more careful approach should be taken when selecting outcome measures for analyses within the selected tests.

### Ecological Validity

The goal of assessment of neuropsychological functions is to predict task performance in real-life settings (37). Therefore, there is great importance in evaluating Ecological Validity—the “*functional and predictive relationship between the patient's performance on a set of neuropsychological tests and the patient's behavior in a variety of real-world settings*” (38)—in neuropsychological research. Traditionally, neuropsychological tests are associated with moderate degree of ecological validity (31), but evidence points to significantly limited ecological validity in psychopathology (19). Indeed, emotional problems in everyday life have been termed “the conditional neurological lesion” (39), and neuropsychology researchers have long recognized that individuals may display intact performance on a task in a quiet room but may show significant difficulties in everyday settings due to the marked impact of psychopathological symptoms on cognitive functions (31). Conversely, assessment settings may provoke anxiety and potentially negatively impact performance, compared to everyday settings where individuals may not feel they are being evaluated. Unfortunately these important situational factors are rarely addressed in the context of neuropsychological studies in psychopathology in general, and in OCD in particular (19). Limited research suggests that this problem is evident in OCD. For instance, despite consistently reported non-verbal memory deficits in OCD (i.e., poor performance on the Rey Complex Figure test), everyday memory functioning in OCD was found to be unimpaired relative to non-psychiatric controls (40). Similarly, in the context of tests of inhibitory function, although suboptimal test performance has been reported in OCD, behavioral impulsivity (the corresponding real-life behavioral construct) in OCD is found to be consistently lower or equivalent compared to non-clinical controls (41). In fact, a study that directly examined performance on different neuropsychological tasks of inhibitory control in OCD found no associations with real-life behavioral impulsivity (42). Another study assessed performance on executive function tasks as well as on a questionnaire of real-life behaviors reflecting executive function [i.e., the Behavior Rating Inventory of Executive Function (BRIEF)] before and after a 14-week CBT treatment in a sample of youth with OCD (43). This study found no change post-treatment on neuropsychological tasks, but a meaningful improvement on real-life functions as assessed by the BRIEF.

In the context of neuropsychological testing, assessment of ecological validity assumes two general approaches, *veridicality*, which is the degree that a neuropsychological test corresponds empirically to outcome measures of everyday function, and *verisimilitude*, which is the degree to which test demands mimic the demands of everyday environments (31). The vast majority of neuropsychological research uses veridicality testing, which is generally known to have modest association with real-life functions (44). For example, the most common tests utilized in OCD research to assess planning are the Tower of London and the Tower of Hanoi tests, in which the primary demand is to copy a structure of beads or discs while adhering to task rules. This test, that involves planning, may be far removed from the real-life demand of planning a vacation for example. Unfortunately, there is a dearth of research into cognitive function in OCD that utilizes tests assuming the verisimilitude approach. These tests may assess complex everyday tasks, such as the Multiple Errands Test [MET; (45)], a test that mimics real-life scenarios related to chores and shopping. Furthermore, with the advancement and availability of virtual reality (VR) technology, verisimilitude tests may become more prevalent in research settings, and in fact may provide a unique integration between veridicality and verisimilitude approaches (46). However, studies that assess cognitive function using VR technology are practically non-existent in OCD. Notably, many of these tests possess very good psychometric properties (47), and researchers are encouraged to consider utilizing such tests to aid in elucidating the nature of cognitive deficiencies in OCD, as well as their relationship to everyday function and psychopathological mechanisms.

## State/Trait Personal Variables

### Correspondence With Clinical and Functional Indices

Despite previous research suggesting that neuropsychological performance may be related to symptom severity, severity has not emerged as a significant moderator of performance on meta-analyses (4, 5, 10). Furthermore, the relationship of test performance to treatment (pharmacological or psychological) is extremely inconsistent (48). While several studies have examined neuropsychological performance as a predictor of treatment outcome (49–52), or in the context of sensitivity to treatment (53–55), results from such studies are extremely sparse and inconsistent, and overall there are no replicable results suggesting that cognitive functions are reliable predictors of response to treatment. This is not surprising, given the lack of associations between neuropsychological test performance and severity measures in the first place.

However, the above inconsistencies and lacunae present a conundrum, as they preclude a meaningful understanding of neuropsychological performance in OCD with regard to psychopathological mechanisms or real-world functioning. Particularly striking is the near-total absence of studies examining correspondence of neuropsychological performance with functional, vocational, and academic indices in OCD, a disorder linked to notable academic and occupational dysfunction (20–22). Moreover, there is little correspondence between neuropsychological assessment of executive function and ratings of real-life functioning (43, 56). This problem however, is not unique to OCD and has been reported across disorders (57, 58) and may partly relate to level of awareness of such difficulties, and the discrepancy between the constructs measured in cognitive tests, and how these are expressed in everyday life. Unfortunately, self-report scales developed uniquely for cognitive difficulties in OCD [e.g., Cognitive Assessment Instrument of Obsessions and Compulsions (CAIOC-13); (59)], have not been examined in relation to neuropsychological test scores.

We recommend that future studies examine the correspondence between neuropsychological performance and functional correlates. This is an essential and highly needed research that would enable the field to learn about the driving factors underlying everyday functional impairments in OCD, and equally important, help to determine what extent of underperformance on cognitive tests may be regarded as indicating real-life functional impairment.

### Affective, Motivational, and Metacognitive Factors

Affective states (e.g., anxiety or depression), motivation, effort, and internal distractions (e.g., intrusive obsessive thoughts) have long been noted as confounds in neuropsychological testing (60), but may carry particular value in explaining discrepant findings. For instance, in some studies, individuals with OCD report greater anxiety about their performance, distracting OCD thoughts, and negative momentary influences during neuropsychological testing (61). In addition, testing of motivation and effort is recommended as an essential part of standard neuropsychological assessments (62), since discrepant performance may indicate sub-optimal effort, attributable to multiple causes including anhedonia, somatization, or secondary gain (19, 63).

Metacognition is the capacity to assess, reflect, control, and evaluate one's cognitions (64). Metacognition is known to be altered across disorders (65). Some aspects of meta-cognition that may impact cognitive function include self-efficacy (66), self-stigma (67), attitudes toward neuropsychological testing (61), and hyper monitoring of one's performance (68). For instance, several explanations of deficient performance in OCD have implicated heightened monitoring of errors or perceived errors, and sensitivity to novelty, including findings regarding post-error slowing on the SST (69, 70), difficulties on simpler/initial test items relative to subsequent/more complex items on the same test (71, 72), and an “always on guard” style of responding even when task demands are relaxed (73). These findings have contributed to the understanding that OCD may be characterized, not by impulsivity, but by over-cautious and inflexible performance monitoring (74–77). Importantly, as depicted by the Executive Overload Model of OCD, such hypercontrol and sensitivity to novel stimuli is related to a surge in obsessive thoughts and may cause an “executive overload” and adversely affect test performance (78).

Other metacognitive processes impacting attention/working memory are evidenced from studies on non-clinical samples—negative expectations relating to task difficulty/own ability (79), stereotype threat (80), rumination and emotional arousal (81), and threat to self-esteem (82). Threat to self-esteem, and lower self-esteem, is posited to affect multiple cognitive functions, including attention through increased state anxiety and (metacognitive) diversion of attentional resources to task-irrelevant stimuli (83). In OCD, stigma/self-stigma from negative stereotypes about cognitive dysfunction in this disorder appear to adversely impact neuropsychological test performance (84). Metacognitive processes, particularly as they relate to self-monitoring and subsequent reframing may also have a facilitatory effect on cognition. Such processes may be utilized to mitigate negative influences on test performance, and assist in selection and use of task-relevant strategies. Hence metacognitive techniques have been included in cognitive remediation interventions (85, 86); however applications to OCD are few, and bear further investigation (87, 88).

Psychological processes impacting test performance are often overlooked and it is recommended that such processes be closely investigated in future studies. Studies so far have employed several approaches to address this issue, such as breaking down test performance to component processes [e.g., (7, 32)], employing experimental modification to classic tasks [e.g., (73)], use of self-reports to assess metacognitive processes during [e.g., (89)], or after [e.g., (61)] neuropsychological task performance. Such research may be crucial to clarifying inconsistencies in findings and improving goodness-of-fit to psychopathological models and real-world correlates of functioning in OCD.

## Discussion

A vast body of literature indicates that OCD is associated with underperformance on neuropsychological tests across multiple domains. However, attempts to integrate cognitive dysfunction with contemporary OCD models or psychopathological mechanisms have been unfruitful, and unexplained heterogeneity remains a major problem. Indeed, moderator analyses across multiple meta-analyses failed to identify any variable or combinations of variables that may account for this heterogeneity. Further attempts to resolve this issue included meta-analyses directly examining moderators, such as symptom severity (10) and OCD dimensions (11, 12), which did not yield meaningful results. In addition, these findings seem to be non-specific to OCD, and such cognitive dysfunction is seen across DSM disorders with very similar effect sizes. Indeed, recently Abramovitch et al. (19) conducted a systematic review of meta-analyses examining cognitive functions across disorders and concluded that psychopathology (defined categorically or dimensionally) is characterized by cognitive dysfunction. This transdiagnostic finding—termed the C Factor (for cognitive dysfunction)—raises the question about common factors across disorders that, like the p factor (90), may have better explanatory power.

However, analyses of moderators that may explain such heterogeneity depend on moderators that researchers choose to assess. These are largely circumscribed to demographic and classic clinical factors. It is important to consider that observable cognitive functioning may be the final product of intricate dynamics involving genetic, neurophysiological underpinnings, neuropsychological functions, psychological factors such as metacognitive biases, and state-related changes in affect and symptoms. Despite mounting evidence, assessment of psychological aspects including motivational and metacognitive factors related to performance is not part of standard neuropsychological research—even though best practice in neuropsychology requires that a conclusion regarding the results of any neuropsychological assessment be made only if effort has been assessed as part of the test battery (62). In particular, the marked inconsistencies in OCD research make assessing these aspects imperative. We recommend that future research consider state/trait personal variables that may impact test performance in OCD, which may also increase interpretive power, and goodness-of-fit with psychopathological models.

Notwithstanding, given that it is becoming increasingly clear that the ecological validity of classic neuropsychological tests in the context of psychopathology (and particularly in OCD) is poor, we recommend that researchers take a careful approach toward selection of tests and outcome measures, as well as with regards to interpretation of their results. Neuropsychological research in OCD would benefit from a careful consideration of tasks and outcome variables, and incorporation of assessment of everyday function is crucial. We also encourage researchers in the field to utilize the verisimilitude approach, incorporating tests that mimic the demands of real-life situations, instead of focusing solely on tests that may be correlated with real-life functions. In addition, self-report systems tapping into real-life functions related to cognitive domains (e.g., the BRIEF) would be of added value. Formation of an international neuropsychological consortium of researchers may be a potential venue to discuss these and other issues, and work toward clearer delineation of suitable tests.

In sum, following decades of exhaustive foundational research on neuropsychology in OCD, subsequent efforts may need to be broader (e.g., consider the role of other factors impacting cognitive dysfunction), deeper (e.g., explore tests and constructs in relation to neuropsychological methods, clinical, and functional correlates), and finer (e.g., undertake more nuanced investigations of test performance), in order to advance the field.

## Author Contributions

All authors listed have made a substantial, direct and intellectual contribution to the work, and approved it for publication.

## Conflict of Interest

The authors declare that the research was conducted in the absence of any commercial or financial relationships that could be construed as a potential conflict of interest.

## Publisher's Note

All claims expressed in this article are solely those of the authors and do not necessarily represent those of their affiliated organizations, or those of the publisher, the editors and the reviewers. Any product that may be evaluated in this article, or claim that may be made by its manufacturer, is not guaranteed or endorsed by the publisher.
